# A State-of-the-Art Survey on Artificial Intelligence to Fight COVID-19

**DOI:** 10.3390/jcm10091961

**Published:** 2021-05-02

**Authors:** Md. Mohaimenul Islam, Tahmina Nasrin Poly, Belal Alsinglawi, Ming Chin Lin, Min-Huei Hsu, Yu-Chuan (Jack) Li

**Affiliations:** 1 Graduate Institute of Biomedical Informatics, College of Medical Science and Technology, Taipei Medical University, Taipei 110301, Taiwan; d610106004@tmu.edu.tw (M.M.I.); tahmina6969@gmail.com (T.N.P.); arbiter@tmu.edu.tw (M.C.L.); 2 International Center for Health Information Technology (ICHIT), Taipei Medical University, Taipei 110301, Taiwan; 3 Research Center of Big Data and Meta-Analysis, Wan Fang Hospital, Taipei Medical University, Taipei 110301, Taiwan; 4 School of Computer, Data and Mathematical Sciences, Parramatta South Campus Western, Sydney University, Sydney, NSW 2116, Australia; b.alsinglawi@gmail.com; 5 Department of Neurosurgery, Shuang Ho Hospital, Taipei Medical University, Taipei 110301, Taiwan; 6 Professional Master Program in Artificial Intelligence in Medicine, Taipei Medical University, Taipei 110301, Taiwan; 7 Graduate Institute of Data Science, Taipei Medical University, Taipei 110301, Taiwan; 701056@tmu.edu.tw; 8 Department of Dermatology, Wan Fang Hospital, Taipei 116081, Taiwan; 9 TMU Research Center of Cancer Translational Medicine, Taipei Medical University, Taipei 110301, Taiwan

**Keywords:** machine learning, deep learning, COVID-19, coronavirus, artificial intelligence

## Abstract

Artificial intelligence (AI) has shown immense potential to fight COVID-19 in many ways. This paper focuses primarily on AI’s role in managing COVID-19 using digital images, clinical and laboratory data analysis, and a summary of the most recent articles published last year. We surveyed the use of AI for COVID-19 detection, screening, diagnosis, the progression of severity, mortality, drug repurposing, and other tasks. We started with the technical overview of all models used to fight the COVID-19 pandemic and ended with a brief statement of the current state-of-the-art, limitations, and challenges.

## 1. Introduction

Coronaviruses are positive-sense single-stranded RNA viruses; they belong to the *Coronavirdiae* family, and mainly infect birds, mammals, and humans [1]. The *Coronavirdiae* family consists of two subfamilies (*Letovirinae* and *Orthocoronavirinae*), and five genera, such as *Alphaletovirus*, *Alphacoronavirus, Betacoronavirus, Gammacoronavirus,* and *Deltacoronavirus* [2]. The coronavirus disease 2019 (COVID-19) is caused by a severe acute respiratory syndrome coronavirus 2 (SARS-CoV-2), which was first introduced in Wuhan, China [3]. The severe acute respiratory syndrome coronavirus (SARS-CoV) and Middle East respiratory syndrome coronavirus (MERS-CoV) are a genus of *Betacoronavirus* and have infected more than 120 million people worldwide [4].

As of 11 February 2021, 107 million confirmed cases have been reported in 212 countries and the mortality rate is 2.19% (https://www.worldometers (accessed on 11 Febuary 2020). There are no specific treatments available for COVID-19 so far, and most of the clinical therapies mainly focus on coping with the symptoms. However, several antiviral drugs, such as remdesivir, were tested and approved for treating severe COVID-19 patients [5,6]. Hhydroxychloroquine, antirheumatic, and angiotensin inhibitors were also used for the management of patients, especially those at high risk [7,8]. Researchers around the world are developing potential vaccines to treat patients that will directly target the virus or block viral entry. Indeed, this pandemic has created a global challenge in many ways, such as increasing demand for medical personnel’s (doctor, nurse, and pharmacists), hospital beds, and medications [9]. The reverse transcriptase–polymerase chain reaction (RT-PCR) is considered as a gold standard tool to identify COVID-19 patients, but the number of RT-PCR is not sufficient, and the diagnostic accuracy is less than eighty percent [10,11]. The availability of standard diagnostic tools for COVID-19 is currently in shortage in the developing countries. This contributes to increased infection rates and delays critical preventive measures.

**Figure 1:**
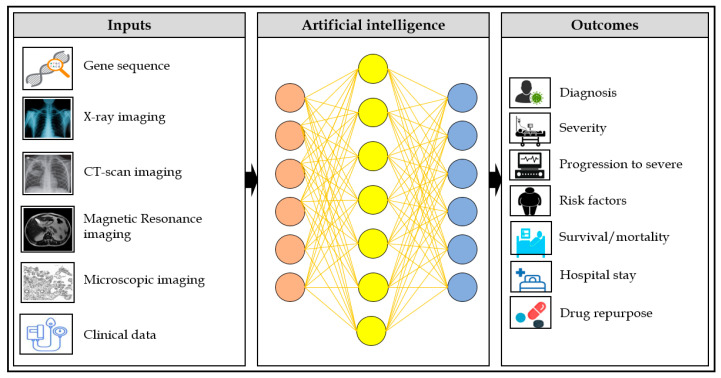
Application of AI to fight COVID-19.

Artificial intelligence (AI) algorithms have shown great potential to predict, diagnose, image classification, and epidemiological trends of diseases. Thus, the application of AI can immediately be applied to combat COVID-19. However, tackling COVID-19 depends on many variables, including rapid diagnosis, screening, accurate stratification of severe patients, and proper treatments. This review shows the contribution of AI models to combat COVID-19 (Figure 1).

## 2. Overview of Artificial Intelligence

The main objective of this section is to provide a formal idea and definition of the artificial intelligence (AI) concepts, techniques and architectures that we found in the COVID-19 related papers surveyed in this study.

### 2.1. Machine Learning

#### 2.1.1. Random Forest (RF)

Random forest (RF) is a simple, diverse, and high performing algorithm introduced by Tin Kam Ho [12]. It is preferred by machine learning practitioners [13]. This algorithm generates multiple decision trees and averages them together to make an accurate prediction [14]. The RF algorithm’s working process is almost similar to boosting; it is easy to train and tune. The main advantage of the RF model is to use both classification and regression tasks. The RF model always utilizes to make correct decision trees and reduce the overfitting problem [15]. Although the RF gives higher performance than decision trees, accuracy cannot outperform gradient boosted trees. In general, bootstrap aggregating or bagging techniques are applied in the RF algorithm to train the model. For example, begging repeatedly (N times) and select a random sample with a replacement from the training dataset, $X=x_{1},\;x_{2}\ldots ,\;x_{n}$ with regarding the outcome $Y=y_{1,\;}y_{2},\;\ldots ,\;y_{n}$. For, i=1,…, N: (a) Random sample, with replacement, *n* train data of *X*, *Y*; express in $X_{i},\;Y_{i}$ (b) train a classification and regression tree $f_{i}$ on $X_{i},\;Y_{i}$. The RF model can be presented in:

$$\hat{y}=\frac{1}{m}\overset{m}{\underset{j=1}{\sum }}\overset{n}{\underset{i=1}{\sum }}W_{j}(x_{i},\overset{´}{x})y_{i}$$

with $W\left(x_{i},\overset{´}{x}\right)=\frac{1}{\overset{´}{k}}$, if $x_{i}$ is one of the $\overset{´}{k}$ point in the same leaf as $\overset{´}{x}$, 0 otherwise.

#### 2.1.2. Support Vector Machine (SVM)

A support vector machine (SVM) is a widely used supervised machine-learning algorithm utilized in classification and regression problems. The SVM algorithm’s main objective is to make a perfect decision boundary or line that can separate n-dimensional space into the various correct category [16]. The most appropriate boundary line with fewer errors is called a hyperplane. The equation for hyperplane can be examined as follows:

$$w_{0}+w_{1}*x_{1}+w_{2}*x_{2}+\ldots +w_{n}*x_{n}=0$$

Let us assume the outcome y (outcomes) is either 1 (yes) or −1 (no). All of these three lines bellows are considered as separating hyperplanes. They are used to separate the outcome y (yes or no) and this property can be presented mathematically as follow:

$$w_{0}+w_{1}*x_{1}+w_{2}*x_{2}>0\;if\;y=1\;\left(yes\right)$$

$$w_{0}+w_{1}*x_{1}+w_{2}*x_{2}<0\;if\;y=-1\;\left(no\right)$$

#### 2.1.3. Logistic Regression (LR)

Logistic regression (LR) is a popular algorithm used to measure the relationship between the dependent variable, such as mortality of patients with COVID-19, and one or more independent variables or predictors (e.g., age, gender, lymphocytes, albumin, LDH, hypersensitive C—reactive protein (hs-CRP)) by calculating probabilities using a logistic function. LR includes a particular group of models named a generalized linear model. It can be explained simply by the following equation:

$$Mortality=\{\begin{array}{c} 1\;\;w_{0}+w_{1}x+\varepsilon >0\\ 0\;\;else \end{array}$$

where ε is an error distributed by the standard logistic distribution. A logistic function is a sigmoid function, which receives any independent variable/predictor (*p*) and gives an outcome value (yes or no); a value between 0 and 1. The standard logistic function σ: ℝ→(0, 1) is presented as bellows:

$$\sigma \;\left(p\right)=\frac{e^{p}}{e^{p}+1}=\frac{1}{1+e^{p}}$$

Suppose, p is a linear function of one independent variable/predictor x. Then p can be presented as follows:

$$p=w_{0}+w_{1}x$$

And the general logistic function l:ℝ→(0,1) is now expressed as:

$$l\left(x\right)=\sigma \left(p\right)=\frac{1}{1+e^{-\left(w_{0}+w_{1}x\right)}}$$

If there are multiple predictor variables for mortality predictions, the expression $w_{0}+w_{1}x$ can be revised to

$$w_{0}+w_{1}x_{1}+w_{2}x_{2}+\ldots +w_{m}x_{m}=w_{0}+\overset{m}{\underset{i=1}{\sum }}w_{i}x_{i}$$

where $w_{m}\left(i\in \left[0,\;m\right]\right)$ are the mortality risk prediction model parameters, m is the number of predictor variables, and $x_{i}$ are predictor variables in a given COVID-19 patient.

#### 2.1.4. XGBoost

XGBoost is an ensemble algorithm that has recently been widely applied to machine learning prediction models because of its speed and performance. This method’s main principle is to boost weak learners to make them strong learners using gradient descent architecture. It helps to minimize a regularized objective function and reduce model complexity. It also trains the dataset iteratively, adding new trees that predict the residuals or errors of prior trees. Lastly, it combines all predictive values of previous trees to make a final prediction.

### 2.2. Deep Learning

#### 2.2.1. Artificial Neural Networks (ANNs)

ANNs are one of the main tools used in AI. ANNs are inspired by the neurons of a biological brain that is intended to mimic how humans learn. ANN consists of input, hidden, and output layers. The input layer is the first layer that receives information in numbers, documents, texts, images, and audio files. The middle layer is called the hidden layer, and a single layer neural network is called a perceptron. However, it can be multiple layers and gives single or multiple outcomes.

**Figure 2:**
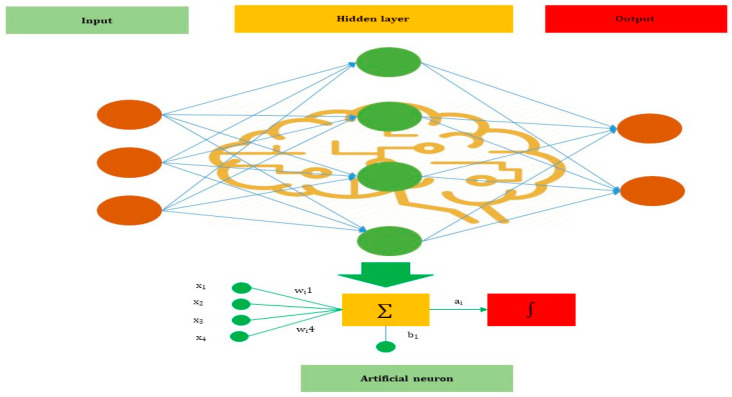
The basic structure of ANN.

In Figure 2*,* $x_{1},$ $x_{2},$ $x_{3}$, and $x_{4}$ represents four inputs (independent variables) to the network. Each of the four inputs is multiplied by a random weight. The weights are represented as $w_{1}$, $w_{2}$, $w_{3}$, $w_{4}$ Weight represents the strength of each node and *b* is called bias. A bias value lets the activation function go up and down. The following output is generated in the activation function:

$$x_{1}\times w_{1}+x_{2}\times w_{2}+x_{3}\times w_{3}+x_{4}\times w_{4}$$

The activation function determines whether a neuron would be activated or not by the sum of weight and further adding bias to it. The primary objective is to introduce non-linearity into the output of each neuron. There are various activation functions used in the neural network, such as:

#### 2.2.2. Convolutional Neural Network (CNN)

**Figure 3:**
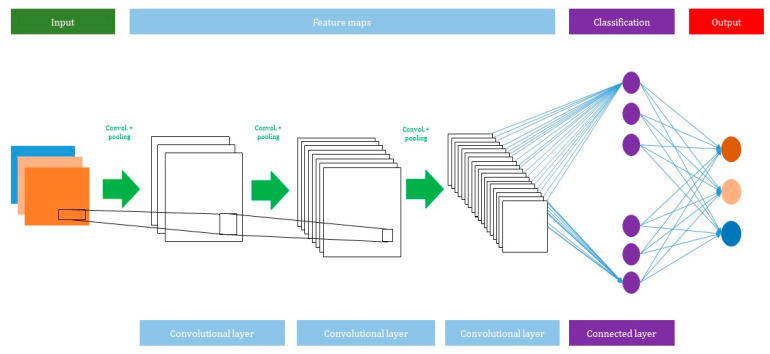
A schematic view of the CNN model.

A CNN consists of several network layers such as input, convolutional, max pooling, average pooling, and output layers. The total number of layers can be increased or decreased based on how many inputs are used to train the model. However, the deeper network will perform better in a large dataset. The advantage of using CNN is that it does not need any feature extraction. In the CNN model, the features are automatically extracted hierarchically from the input, and it is further classified by using a fully connected layer. Figure 3 shows the architecture of the CNN model.

**Convolutional layer**: A convolutional function is applied in the convolutional layer to use given input variables. A filter moves over the input variables with a stride (describes how many pixels per filter will be translated horizontally and vertically). The providers usually determine the size of the stride. It generates feature maps and is used as an input of the subsequent layer.

**Activation function**: Different types of activation function are applied in the convolutional layers. It helps to create a non-linear relationship between the data and the output class.

Layer l is a non-linearity layer and it takes the feature volume $Y_{I}^{\left(L-1\right)}$ from a convolutional layer (l−1) and generates the activation volume $Y_{i}^{\left(l\right)}$.

$$Y_{i}^{\left(l\right)}=f\left(Y_{i}^{\left(l-1\right)}\right)$$

There are several types of activation function, such as tanh, sigmoid, and ReLu, used to classify output variables. However, ReLu is a widely used activation function because of its capability to reduce the exploding/vanishing gradient problem.

Tanh: f(x)=tanh(x)

$$Sigmoid:\;f\left(x\right)=\frac{1}{1+e^{-x}}$$

ReLu: f(x)=max(0,x)

**Figure 4:**
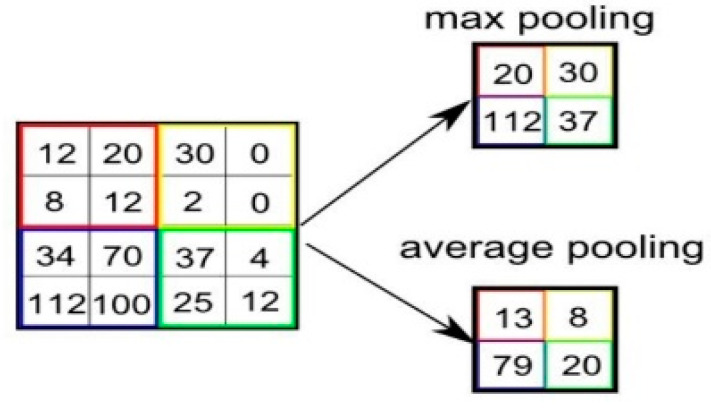
Max pooling in CNN.

**Max pooling**: A max pooling layer is used to reduce the size of the feature. The value of stride is selected according to the maximum value/average value (Figure 4). The maximum/average value is taken by stride and a matrix is made. However, the output size of the layer is smaller than the previous layer.

**Figure 5:**
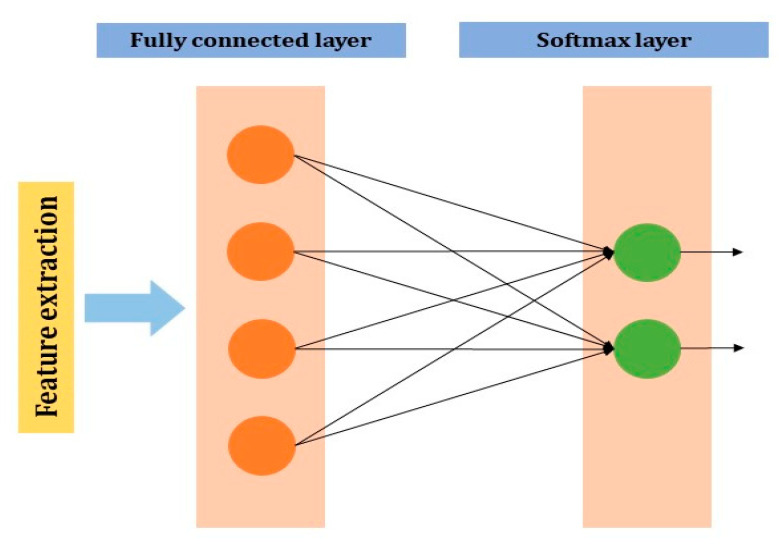
Fully connected layer in CNN.

**Fully connected layer**: The neuron of the previous layer i.e., max pooling layer will be connected to each and every neuron in this layer. The output layer of the MLP will have $m_{1}^{\left(l-i\right)}$ outputs. In the output neurons, i denotes the number of layers in the MLP (Figure 5).

If l−1 is a fully connected layer;

$$y_{i}^{\left(l\right)}=f\left(z_{i}^{\left(l\right)}\right)\;\mathrm{with}\;z_{i}^{\left(l\right)}=\sum_{j=1}^{m_{1}^{\left(l-1\right)}}w_{i,j}^{\left(l\right)}y_{i}^{\left(l-1\right)}$$

#### 2.2.3. Neural Recurrent Network (RNN)

RNN is a generalization of a feedforward neural network that uses information sequentially. Traditionally, all inputs (and outputs) of the neural network are considered independent of each other. The RNN performs the same task for every input of data, and the output always depends on the previous computations. Every time the output is made, it is then copied and sent back to the recurrent network. To make the final output, it takes the current input and the output that it has already learned from the previous input of data. Unlike feedforward neural networks, RNNs have an internal state (memory) that can process sequences of inputs and capture input and output information about what has been calculated. This ability makes RNN applicable to real-time clinical decision-making tasks.

**Figure 6:**
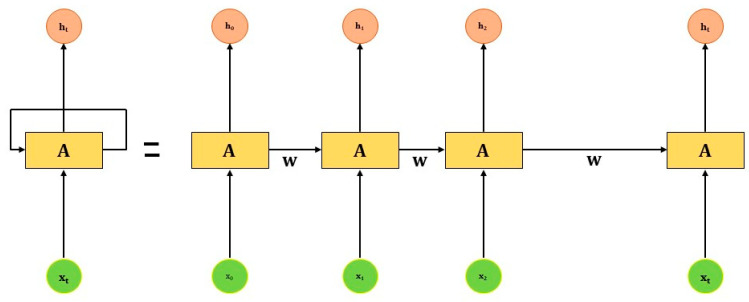
The architecture of RNN.

In Figure 6, it shows that RNN takes input $x_{0}$ from the sequence of data and makes an output ($h_{0}$), which then combines with another input $x_{1}$ for the next step. Therefore, the next input is a combination of the output of the previous input $h_{0}$ and second input $x_{1}$. Similarly, the next input will be a combination of output $h_{1}$ and input $x_{2}$, and so on. In this process, RNN continues to remember the information while training.

The mathematical equation for the current state and activation function is given below:

$$h_{t}=f(h_{t-1}\;,\;x_{t})$$

$$h_{t}=\tan h(w_{hh}h_{t-1}+w_{xh}x_{t}$$

where W is weight, h is the single hidden vector and $w_{hh}$ is the weight at a previous hidden state, $w_{hx}$ is the weight at the current input state, tanh is the activation function that converts input to range (–1,1), and output state $y_{t}$ is produced by $y_{t}=w_{hy}h_{t}$ where $w_{hy}$ is the weight at the output state.

#### 2.2.4. Long Short Term Memory (LSTM)

**Figure 7:**
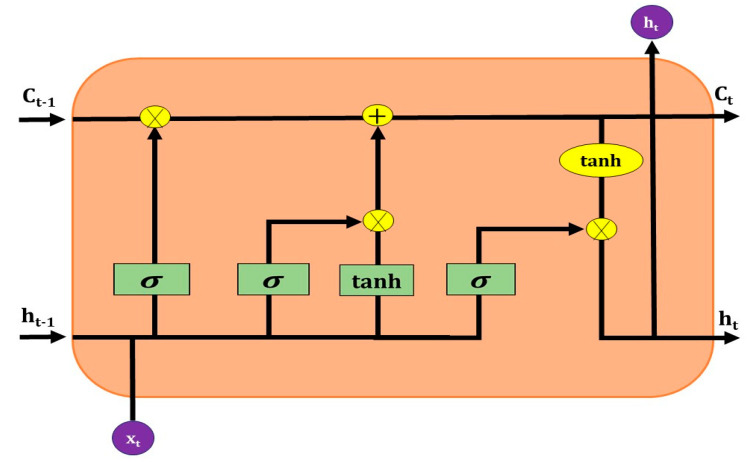
A basic structure of LSTM.

LSTM is a modified version of RNN, which can easily collect data information in the memory cell. LSTM is applied to overcome the vanishing gradient problems through a gating mechanism and is more applicable to classify processes and predict real-time clinical problems given time lags of unknown duration. The primary tool of LSTM’s is the cell state (memory) and its’ various gates. The cell state of LSTM transfers valuable information to the whole sequence. Adding useful information from previous time steps can make its way to later time steps, minimizing the impact of short-term memory. Since the cell state proceeds forward, potential information is added or deleted to the cell state via gates. The gate decides what information is allowed on the cell state, and the cell state determines what information is valuable to retain or delete during the training process. LSTM consists of input, forget, and output gate (Figure 7).

**(a) Input gate**: The input gate used to update the cell state; it helps to pass the previous *hidden state* and current input into a sigmoid function. The sigmoid activation function transforms the values between 0 and 1. The value closer to 0 means unimportant, and closer to 1 means important. The input gate also passes the same hidden state and current input into a tanh function to convert values between −1 and 1.

Finally, the output from the sigmoid function would multiply with output from the tanh function; however, the sigmoid output makes a decision on what information could be retained from the tan*h* output.

$$i_{t}=\sigma \;\left(W_{i}.\left[h_{t-1},\;x_{t}\right]+b_{i}\right)$$

$$C_{t}=\tan h(W_{C}.[h_{t-1},x_{t}]+b_{C})$$

**(b) Forget gate**: the forget gate decides which information can be retained or deleted. Values from the previous hidden state and current input go through the sigmoid function and convert them between 0 and 1. The value closer to 0 omits, and closer to 1 retains.

$$f_{t}=\sigma \left(W_{f}.\left[h_{t-1},x_{t}\right]+b_{f}\right)$$

**(c) Output gate**: The output gate makes a decision on what the next hidden state would be and the hidden state stores all valuable information on previous inputs, which are eventually used to predict. In this process, the previous hidden state and the current input goes through a sigmoid function (convert value 0 and 1), and the newly modified cell state goes through the tanh function (convert value −1 and 1). Storing valuable information in the hidden state is decided by multiplying the tanh output with the sigmoid output. The new cell state ($C_{t}$) and the new hidden state ($h_{t}$) is then transferred over the next step.

$$O_{t}=\sigma \left(W_{0}\left[h_{t-1},x_{t}\right]+b_{o}\right)$$

$$h_{t}=O_{t}\times \tan h\left(C_{t}\right)$$

## 3. Review of State-of-the-Art

AI has been applied to many areas of COVID-19, including screening, diagnosis, severity stratification, mortality prediction, and epidemiology controls. A review of the related recent state-of-the-art is shown below:

### 3.1. COVID-19 Screening Using Digital Images

#### 3.1.1. Potentiality

The prevalence of COVID-19 has been increasing, and the healthcare industry is facing a lack of healthcare providers to handle this unprecedented situation. Digital images, such as X-ray and CT scan, have been used to check abnormalities and stratify COVID-19 patients. Recently, deep learning shows its’ capabilities to detect disease accurately using digital images. Therefore, developing AI systems could help physicians to screen COVID-19 patients efficiently and lessen the burden on hospitals dealing with outbreaks. Kumar et al. [17] developed a machine learning-based classification model using a deep feature for COVID-19 patient’s prediction. The XGBoost model showed higher classification performance (AUROC: 0.99, accuracy: 0.97, sensitivity: 0.97, and specificity (0.98)) than other models. Minaee et al. [18] trained 2000 X-ray images using convolutional neural networks, including ResNet18, ResNet50, SqueezeNet, and DenseNet-121 to stratify early COVID-19 patients and achieved a sensitivity of 98%. Moreover, Karim et al. [19] developed an explainable deep learning prediction model using 15,959 chest radiography images from three categories of patients (COVID-19, normal and pneumonia). While evaluating their model, they achieved precision, recall, and an F1 score of 0.904, 0.905, and 0.905, respectively. Table 1 shows several deep learning-based prediction models for COVID-19 patients’ classification.

#### 3.1.2. Limitations 

The primary objective of the AI model is to stratify COVID-19 patients from healthy patients. CNN model showed an efficient performance to be considered in the real-world clinical setting. However, there are several potential limitations. First, all of the studies were poorly reported and had a high risk of bias, therefore, considering their findings in the real-world clinical setting would be optimistic. Second, all of the studies had a lack of external evaluation and most of the studies used similar datasets, which raised potential bias. Third, no study mentioned where to deploy and how to interpret their findings; therefore, there is a lack of applicability information. Fourth, only a few studies reported a positive and negative predictive value, but these metrics are important to make a clinical decision using the findings in the hospital settings.

### 3.2. Artificial Intelligence for COVID-19 Severity

#### 3.2.1. Potentiality

A large number of patients being hospitalized due to COVID-19, and mortality risk is also high. Previous studies reported that approximately 30 percent of patients go to the ICU among hospitalized patients, and 12–33 percent of patients need mechanical ventilation supports [30,31,32]. Identifying predictors for disease severity would help the physician make a crucial decision on which patients’ group needs to be prioritised or treated sooner (Table 2). Cai et al. [32] used CT images of COVID-19 patients to develop a prediction model that stratifies disease severity into groups (moderate, severe and critical). Lassau et al. [33] developed an AI-based scoring system to predict disease severity using just five clinical and biological variables. The performance of their model slightly increased while adding radiology variables. Moreover, Yip et al. [34] used 107 radiomic features of 1110 COVID-19 patients for developing a prediction model. Their model successfully stratified severe patients from mild patients with an AUROC of 0.85. Some symptoms (e.g., dyspnea, respiratory rate, heart rate and comorbidities (e.g., cardiovascular disease, hypertension, and diabetes)) significantly differed between mild and severe patient. A combination of clinical variables and radiological variables provided better performance. For example, a radiological feature such as ground-glass opacity significantly impacted severity prediction [35]. Quiroz et al. [36] aimed to develop an AI-based prediction model using clinical and imaging data from 346 patients. Different modern machine learning techniques, including, XGBoost, were used and several essential features also identified. The logistic regression model outperforms other models and accurately differentiates mild and severe patients (AUC 0.950; sensitivity 0.764; specificity 0.919).

#### 3.2.2. Limitations

AI models showed potentially significant performance in accurately predict the prognosis of the COVID-19 disease after hospitalization. However, these studies had several limitations. First, they had a small sample size, therefore the interpretation of results could be varied. Second, all of the studies collected data retrospectively and their results could be different while using their findings prospectively in the real-world clinical setting. Third, the range of missing data varied among studies; therefore, some potential information might be missed during the severity prediction. Finally, it is unclear what the optimal number of variables to be used is, and what kinds of variables (only laboratory and CT or a combination of both etc.) should be used to predict the disease progression during admission.

### 3.3. Artificial Intelligence for COVID-19 Mortality

#### 3.3.1. Potentiality

COVID-19 has already shown its’ fatality, and the number has been increasing each day. Healthcare providers are struggling to make effective decisions for severe patients. This is because the pattern of the disease is complex. However, AI is an effective tool to predict the mortality of COVID-19 using a large number of clinical, laboratory, and image data. Timely grouping of the high-risk patients can help the physician make valuable medical decisions on who needs to receive more attention [44]. Several studies attempted to make an effective personalized mortality risk scoring system and evaluated their model with a new dataset [45,46] (Table 3). Booth et al. [47] demonstrated that serum biomarkers such as C—reactive protein (CRP), blood urea nitrogen (BUN), serum calcium, serum albumin, and lactic acid are significantly associated with an increased risk of mortality. Similarly, Zhou et al. [48] identified several risk factors (serum ferritin, procalcitonin, and CRP) that are directly associated with increased risk of severity and mortality. Moreover, epidemiological and clinical variables were significantly different between the survival and mortality group [49]. For example, the mortality rate was higher in patients with older age, obesity, and cardiac diseases [50,51].

#### 3.3.2. Limitations

Although, the overall performance of the AI model to predict COVID-19 mortality was satisfactory in terms of sensitivity and specificity. However, there were several potential limitations, namely generalizability (it had limited external validation), single-site study, and a small minority of cohort. The majority of the studies did not include important variables such as hematological, biochemical, radiological, and microbiological variables. Moreover, different studies used different feature selection models; therefore, the number of variables to predict COVID-19 mortality is fixed, such as conventional scoring systems. Finally, most of the study failed to show the effect of drugs on mortality risk prediction. Some studies used drug history as input variables, but they did not mention how long the patients had taken the medications, and there was no information about the dose.

### 3.4. Artificial Intelligence for COVID-19 Drug Repurposing

#### 3.4.1. Potentiality

There is no specific treatment available to fight COVID-19, and the development of new drugs needs 10–15 years [62]. A previous study demonstrated that approximately 30–40% of total drugs were used for a new purpose; although, drug repurposing is sometimes performed accidentally or in a limited way [63]. A recent technological advancement has opened opportunities for the systemic evaluation of any drug indication against new diseases. Drug repurposing not only saves time and costs, but it ensures patient safety because potential adverse effects have already tested. Text mining and bioinformatics tools are used to screen biological and chemical data to find known drugs against an array of COVID-19 target. The drug-repurposing strategy was used previously to combat several life-threatening diseases; therefore, finding its role to tackle the COVID-19 pandemic could be a great option. Cantürk et al. [64] utilized a neural network model to discover the underlying associations between viral proteins and antiviral therapeutics, which can be used to treat COVID-19 patients. AI researchers are very enthusiastic about drug repurposing to fight COVID-19 and have already proposed AI methods for searching existing drugs that have antiviral activity against COVID-19 (Table 4).

#### 3.4.2. Limitation

All of the studies showed a promising performance to find several drug candidates that might help to fight agonist COVID-19. However, their findings were not tested experimentally and clinically, which is the most potential limitation. Moreover, their studies are based on the previous knowledge that all of the potential candidates had a strong inhibitory effect on MERS and SARS-CoV; it was not guaranteed that these candidates could strongly fight against SARS-CoV-2. We know that there are lots of variants of SARS-CoV-2; therefore, it is uncertain whether they will be effective against all variants of SARS-CoV-2.

### 3.5. Artificial Intelligence for Epidemic Trends

Since the COVID-19 infection rate was increasing rapidly, it was critically important to predict the development and spread of the epidemic. Wang et al. [77] developed a forecasting model using LSTM, a deep learning algorithm, which was able to predict the rising trend of infection rate in the next 30 days. Their model successfully shows the epidemic trend of COVID-19 infection for Russia, Peru and Iran.

## 4. Overall Challenges to Deploy AI Model in the Clinical Settings

Over the last decade, AI techniques have been showing their ability to predict various diseases. However, the applications of AI in the healthcare industry is now contributing in multiple ways, including decision supports. Although the performance of AI to fight against COVID-19 is promising, there are still many challenges ahead while considering how to deploy these AI models in real-world clinical settings. Several challenges of AI have given bellows:

1. The number of participants used to train the AI models to predict disease progression, mortality risk was not sufficient to deploy in real-world clinical settings. It is a great challenge to train the model using a large number of patients from multiple sites or countries and make the AI model more generable and trustworthy;
2. As all of the studies used different types (laboratory, symptoms, biochemical, CT/X-ray) and a various number of variables to predict the risk of severity and mortality; therefore, it is challenging to establish what kinds of variables should be used, and what the optimal number to be utilized is while admitting COVID-19 patient to the hospital. The traditional scoring systems for stratifying patients have a fixed number of variables, but deciding the fixed number of variables from those studies may be difficult;
3. Making strong evidence and the simplicity of prediction models is also challenging to fight against COVID-19. All of the included studies used different data sets, and the ethnicity was also different. Moreover, they reported a different time frame while predicting disease progression and mortality risk. All of the studies should provide a standard time frame, such as 24 h, 3 days, and 7 days to predict patient’s situation;
4. Generalizability is another potential challenge to deploy the AI model in the real-world clinical setting to tackle COVID-19. The findings of one study might be different while testing it using other datasets from different countries;
5. Reducing bias, such as patient selection, reference standard, and methodology, would be challenging. However, all of the upcoming studies should follow standard guidelines (e.g., TRIPOD) while reporting their findings;
6. Resolving the “black-box” issue would be more challenging; however, all of the studies should provide a clear explanation of predictors and how these predictors influence the performance. They should report univariate and multivariate analysis while showing the performance metrics. Moreover, they should categorize the variables (e.g., symptoms, laboratory, and radiology) and present the model performance for each category;
7. Others (Recommendation from organizations, establishing trust among healthcare providers, decreasing false positive and negative results, and ethical issues).

## 5. Conclusions

The application of AI in pandemic control has shown great potential in various ways, including predicting epidemic trend, patient tracking, stratifying asymptomatic patients, and finding potential repurpose drugs. All of the studies had a lack of sample size, and external validation and inappropriate model evaluation; therefore, using these findings would be an optimistic decision. The finding of our study does not suggest using these prediction models for diagnosis, disease progression, and mortality risk. However, future research with a large sample size and proper interpretation could be evaluated using multiple datasets before considering these in the real-world clinical setting. Moreover, repurposed drug candidates can also be assessed by clinical experiments. Additionally, studies are needed to assess the actual effectiveness of AI models and calculate the cost-effectiveness in clinical practice. To get the real taste of AI to fight COVID-19, it is essential to reduce the false positive and negative rate as well as to disclose the ‘black-box’ nature of AI.

## Figures and Tables

**Table 1:** The performance of AI model for COVID-19 detection.

Author	Model	Algorithms	Applications	Modality	F-1 Score	AUROC/Accuracy
Hemdan [20]	CNN	DenseNet	Classification of COVID-19 and normal	X-ray	0.91	-
Civit-Masot [21]	CNN	VGG16	Classification of COVID-19, Pneumonia, and healthy	X-ray	0.91	>90
Elaziz [22]	CNN	FrMEMs	Classification of COVID-19 and healthy	X-ray	-	--/96 and 98
Wang [23]	CNN	Xception + SVM	Classification of COVID-19 and normal	X-ray	-	99.33/99.32
Das [24]	CNN	VGG-16	Classification of COVID-19, Pneumonia and normal	X-ray	0.96	--/97.67
Kassani [25]	CNN	DesnseNet121+Bagging	Classification of COVID-19 and normal	X-ray and CT scan	0.96	--/99
Ardakani [26]	CNN	ResNet-101	Classification of COVID-19 and normal	CT scan	1.0	99.4/99.5
Jain [27]	CNN	ResNet101	Classification of COVID-19 and viral pneumonia	X-ray	0.98	--/98.15
Singh [28]	CNN	MODE-based CNN	Classification of COVID-19 and normal	CT scan	--	--/93.3
Ahuja [29]	CNN	ResNet 18	Classification of COVID-19 and normal	CT scan	0.99	99.65/99.4

Note: CNN: Convolutional Neural Network.

**Table 2:** The performance of AI model to predict disease severity of patients with COVID-19.

Author	Methods	Application	Variable Types	Precision/Recall	AUROC/Accuracy
Akbar [37]	GBM	Severity of COVID-19	Blood	0.91/0.88	89/89
Feng [38]	RNN	Severity	CT scan	--/0.81	90/94
Xiao [39]	CNN	Severity	CT scan	--/--	89/81.9
Wu [40]	LR	Severity	CT and laboratory	0.66~0.95/ 0.75~0.96	84~93/ 74.4~87.5
Li [41]	CNN	Severity	CT and laboratory	0.82/0.79	93/88
Kang [42]	ANN	Severity	CT, clinical and laboratory	--/--	95/--
Ho [43]	CNN	Severity	CT	0.78/0.80	91/93

Note: CNN: Convolutional Neural Network; RNN: Recurrent Neural Network; ANN: Artificial Neural Network; GBM: Gradient Boosting Method; CT: Computed Tomography.

**Table 3:** The performance of AI model to predict mortality of COVID-19.

Author	Methods	Application	Variable	Sensitivity/Specificity	AUROC/Accuracy
Abdulaal [52]	ANN	Mortality risk	Demographic, comorbidities, smoking history, and symptom	0.87/0.85	-/86.25
An [53]	SVM	Mortality risk	Demographics, symptom, comorbidities, and medications	0.92/0.91	96.3/-
Gao [54]	Ensemble model	Mortality risk	Demographics, comorbidity and vital sign	0.32~0.45/ 0.97~0.99	92~97/ 93.0~95.6
Hu [55]	LR	Mortality risk	Demographic and laboratory	0.83/0.79	88/-
Li [56]	ANN	Mortality risk	Demographics, symptoms and laboratory	0.75/0.87	84/85
Yan [57]	XGBoost	Mortality risk	Demographic, symptom, and laboratory	1/-	92.2~95.05/
Rechtman [58]	XGBoost	Mortality risk	Demographics, symptoms, comorbidities	-	86/-
Ryan [59]	XGBoost	Mortality risk	Demographic, comorbidity, vital sign, and laboratory	0.82/0.84	91.0/80
Vaid [60]	XGBoost	Mortality risk	Demographic, comorbidity, vital sign, and laboratory	-	68~98/
Yadaw [61]	XGBoost	Mortality risk	Demographics, comorbidity, smoking	-	-/91

Note: LR: Logistic Regression; SVM: Support Vector Machine; ANN: Artificial Neural Network.

**Table 4:** Application of AI for COVID-19 drug repurposing.

Author	Application	Model	Data	Results
Beck-2020 [4]	Identifying available drugs that could act on viral proteins of SARS-CoV-2 using Molecule Transformer-Drug Target Interaction (MT-DTI)	Transfer learning and molecular docking	Drug Target Common (DTC) database and BindingDB	antiviral drugs such as lopinavir/ritonavir had been identified by the MT-DTI model should be considered
Choi-2020 [65]	Finding approved drugs that can inhibit COVID-19 by using g a deep learning-based drug-target interaction model called Molecule Transformer-Drug Target Interaction (MT-DTI)	Transfer learning and molecular docking	DrugBank and ZINC	Identified 30 drugs that have strong inhibitory potencies to the angiotensin converting Enzyme 2 (ACE2) receptor and the transmembrane protease serine 2 (TMPRSS2).
Esmail-2020 [66]	Identifying antiviral therapeutic targets for drug repurposing by using the DeepNEU stem cell-based platform and validated computer simulations of artificial lung cells.	Hybrid deep-machine learning system with elements of fully connected RNNs, CMs, and evolutionary systems (GA)	DeepNEU database plus important information upgrades in the form of a new gene, protein, and phenotypic relationship data.	To improve preparedness for and response to future viral outbreaks.
Gusarov-2020 [67]	Identifying potential drugs for SARS-CoV-2 using machine learning algorithms	Machine learning algorithms	N/A	Short for conductor-like screening model for real solvents might assist to accelerate drug discovery for the treatment of COVID-19
Hooshmand-2020 [68]	Finding potential drugs that can inhibit COVID-19 using the Multimodal Restricted Boltzmann Machine approach (MM-RBM)	Multimodal Restricted Boltzmann Machine approach (MM-RBM)	Harmonizome and Literacy Information and Communication System (LINCS)	MM-RBM has immense potential to identify the highly promising medications for COVID-19 with minimum side effects.
N. Ioannidis-2020 [69]	Identifying COVID-19 drugs for repurposing using deep graph learning	RGCN and state-of-the-art KGE	IMDB, DBLP and drug-repurposing knowledge-graph (DRKG)	Their model showed promise to identify possible drug candidates.
Ke-2020 [70]	Identifying the marketed drugs with potential for treating COVID-19 using artificial intelligence method	Deep Neural Network (DNN)	DrugBank,	Identified 80 potential drugs that have the ability to fight coronavirus.
Kowalewski-2020 [71]	Searching several drug candidates for COVID-19 using machine learning algorithms.	Support vector machine	ZINC, ChEMBL 25, DrugBank, EPI Suite, Therapeutic targets databases, Hazardous substances data Bank	Suggested several drugs for repurposed that suited for short-term approval, and long-term approval need follow-up
Loucera-2020 [72]	Aimed at using machine learning models to identify appropriate drugs fight against SARS-CoV-2 infection	Machine learning	DrugBank	It shows promising results and found several drugs that can be considered only a subset of the potential drug candidates for repurposing.
Mohapatra-2020 [73]	Developed a machine-learning model to find drugs already available in the market; can be used for inhibiting SARS-CoV-2 infection.	Classification models such as Naïve Bayes, molecular docking	PubChem Bioassay, DrugBank	The findings suggested that machine-learning algorithms can be identified and tested the therapeutic agents for COVID-19 treatment.
Pham-2020 [74]	Identifying strong associations among biological features, and outputs to predict gene expression profiles given a new chemical compound.	DeepCE based on linear models, vanilla neural network, k-nearest neighbor, and tensor-train weight optimization models.	L1000 gene expression gene, STRING, DrugBank, Gene Expression Omnibus	DeepCE helps to accelerate compound screening against a single target.
Verma-2020 [75]	To evaluate potential response of existing antiviral drug candidates against SARS-CoV-2 target proteins that help viral entry and replication into the host body.	Bayesian machine learning	PubChem, ZINC, DrugBank,	Their model identified 45 drugs that can inhibit SARS-CoV-2. Those drugs work on the major target proteins such as spike protein (S) and main proteases.
Zeng-2020 [76]	To develop a network-based deep-learning method of identifying drugs to work as repurpose drugs for COVID-19	DGL-KE developed by AWS AI	PubMed, DrugBank	Their model identified 41 repurpose drugs that may accelerate therapeutic response against COVID-19

## Data Availability

N/A.
